# Regional Anesthesia for Lower Limb Burn Wound Debridements

**DOI:** 10.5812/atr.6833

**Published:** 2012-10-14

**Authors:** Indu M Sen, Ramesh K Sen

**Affiliations:** 1 Department of Anesthesia and Intensive Care, Post Graduate Institute of Medical Education and Research, Chandigarh, India; 2 Department of Orthopedic Surgery, Post Graduate Institute of Medical Education and Research, Chandigarh, India

**Keywords:** Anesthesia, Burn, Wound Debridements

*Dear Editor,*

We read with interest the article by Rimaz et al.(1). However, there are few queries. We would like to know about the duration of burn injuries. Pain threshold can vary among patients suffering from acute burns posted for first debridement before eschar formation in comparison to patient posted for second or third debridement (2). Similarly, a patient with previous exposure to anesthesia is expected to be more familiar to the setting and the use of Patient Controlled Analgesia (PCA) devices. It appears unethical to not provide any pain relief for patients with up to 35% of total body surface area burns (3). As a result, all the patients excluding patients with daily intake of analgesics, need to be justified. Alternatively, withholding the night dose of analgesic would have sufficed. All the patients enrolled in the study were ASA I or II with lower extremity burn injuries. What was the position of the patient during surgery? In hemodynamically stable, compliant patients, the procedure could have been safely performed under regional anesthesia i.e. a combination of low-dose spinal with lumbar epidural anesthesia or a regional block with adjuvants can be tried for long-term pain relief (4). This could further be supplemented with intravenous sedation and analgesia, a cost-effective alternative of general anesthesia. Moreover, continuous infusion of local anesthetics along with adjuvants via epidural or peripheral route are suggested to be used for postoperative pain relief. Another advantage of this technique is the reduced incidence of postoperative nausea and vomiting. While using regional anaesthetic regimen, preoperative administration of oral gabapentin tablet would lead to reduced postoperative morphine consumption for analgesic purpose. Abstract reads that pain was assessed on a visual analogue scale at rest and during movement at different time intervals before the operation. Please correct this statement to 1, 4, 8, 12, 16, 20 and 24 h after the operation.
